# Comparison of Isoenzyme Pattern of *Echinococcus granulosus* *sensu stricto* (G1-G3) and *E. canadensis* (G6/G7) Protoscoleces

**DOI:** 10.52547/ibj.3815

**Published:** 2023-04-17

**Authors:** Majid Dousti, Seyed Mahmoud Sadjjadi, Rahmat Solgi, Arghavan Vafafar, Yosef Sharifi, Amirhossein Radfar, Gholam Reza Hatam

**Affiliations:** 1 Students Research Committee, School of Medicine, Shiraz University of Medical Sciences, Shiraz, Iran;; 2 Department of Parasitology and Mycology, School of Medicine, Shiraz University of Medical Sciences, Shiraz, Iran;; 3 Department of Medical Microbiology, Birjand University of Medical Sciences, Birjand, Iran;; 4 Basic Sciences in Infectious Diseases Research Center, Shiraz University of Medical Sciences, Shiraz, Iran

**Keywords:** Echinococcus granulosus, Genotype, Isoenzymes

## Abstract

**Background:**

Different genotypes of *Echinococcus granulosus s.l. *infect humans and ungulate animals, causing cystic echinococcosis. Simultaneous isoenzyme, as well as molecular characterizations of this parasite, has not yet been investigated in Iran. The present study aimed to evaluate the isoenzyme pattern of the *E. granulosus s.s. *and *E. canadensis* genotypes in Iran.

**Methods:**

A total of 32 (8 humans and 24 animals) cystic echinococcosis cysts were isolated from Shiraz, Tehran, Ilam, and Birjand from May 2018 to December 2020. The DNAs were extracted and their genotypes were determined by molecular methods. Enzymes were extracted from the cysts and subjected to polyacrylamide gel electrophoresis. The activities of G6PD, MDH, ME, NH1, and ICD were examined in the cyst samples using isoenzyme method and compared it with the genotyping findings.

**Results:**

DNA sequence analysis of the samples showed that the specimens contained 75% *E. granulosus s.s.* (G1) and 25% *E. canadensis* (G6) genotypes. The isoenzyme pattern of ICD in both genotypes produced a six-band pattern with different relative factors. The G6PD also produced two bands with different relative migrations in both genotypes. The MDH and NH1 systems revealed a two-band pattern, while only one band was generated in the ME enzyme in the *E. granulosus s.s.* genotype. In the *E. canadensis*, the MDH and NH1 enzymes showed one band, and the ME enzyme represented a two-band pattern.

**Conclusion:**

Our findings suggest that *E. granulosus s.s.* and *E. canadensis* genotypes have entirely different isoenzyme patterns for NH1, G6PD, MDH, and ME.

## INTRODUCTION

Cystic echinococcosis is a cosmopolitan parasitic zoonosis caused by *E. granulosus s.l.*^[^^1^^]^. Globally, CE is ranked second on the list of food-borne parasitic diseases and also among the 17 most neglected tropical diseases prioritized by the WHO^[^^2^^]^. According to WHO report^[^^2^^]^, more than one million people are affected by echinococcosis at any given time^[^^3^^]^.

The definitive host of *E. granulosus* is domestic dogs, other canids, or even lion. A wide range of ungulates, including sheep, goat, cattle, and camel, plays as intermediate host. Humans acts as aberrant, dead-end intermediate hosts^[^^3^^]^. Contaminated food and drinking water or exposure to the contaminated environment are likely the sources of infection for humans^[^^3^^]^. CE is widespread and common in different regions of the world, especially the Middle East, India, South America, and Australia^[^^4^^]^. It is very important to study different aspects of the disease since Iran is one of the endemic regions, with ~1% of its total surgeries related to this disease^[^^5^^]^.

There is much heterogeneity in* E. granulosus* *s.l.* due to the presence of intraspecific variants or strains^[^^6^^,^^7^^]^. Hence, further identification of *E. granulosus* genotypes and understanding the molecular and biochemical differences is of great importance in designing diagnostic, therapeutic, prevention, and control strategies^[^^8^^]^. In view of phenotypic and molecular studies, *E. granulosus s.l. *is divided into *E. granulosus* *s.s.* (including the identified genotypic variants G1-G3), *E. equinus* (the ‘horse strain’, genotype G4), *E. ortleppi* (the ‘cattle strain’, genotype G5), *E. canadensis *(G7/G7), and *E. felidis* (the former ‘lion strain’)^[^^9^^]^. 

Considerable molecular studies on CE have been carried out in Iran during recent years^[^^6^^,^^10^^-^^15^^]^. Based on the previous studies, *E. granulosus* *s.s.* and *E. canadensis* have been considered prevalent genotypes in different parts of Iran^[^^6^^,^^12^^,^^16^^,^^17^^]^. Moreover, a number of protein studies related to *E. granulosus* have been conducted in recent years in Iran^[^^15^^,^^18^^-^^21^^]^. The present study aimed to investigate the isoenzyme pattern of the larval stage of *E. granulosus* *s.s.* and *E. canadensis* genotypes and enzyme pattern changes within each one of these genotypes and between these two genotypes in Iran. As there is no phenotypic study on the isoenzyme pattern of the *E. granulosus* parasite in Iran, the current research work is the first in this field. 

**Sample collection**

 CE cysts were isolated from the Central, Western, Eastern, and Southern parts of Iran. Animal specimens (sheep, cattle, and camels) were collected from Shiraz, Ilam and Birjand industrial slaughterhouses during May 2018 through December 2020. Human CE cyst were isolated from different hospitals in Shiraz and Tehran and transferred to the Parasitology Laboratory at the Medical School, Shiraz University of Medical Sciences. Following the centrifugation of all samples, a total of 30-100 µl of deposited protoscoleces were removed and washed three times with PBS. The obtained sediments were finally kept at -21 °C for subsequent experiments. 

**DNA extraction**

DNAs were extracted from all collected samples using a DNA extraction kit (Bioneer Company, South Korea) following the manufacturer's instructions.

**PCR method**

 To synthesize fragments selected from the mitochondrial genome of the parasite, JB3 (5'-TTTTTTGGGCATCCTGAGGTTTAT-3') and JB4.5 (5'-TAAAGAAAGAACATAATGAAAATG-3') primers were used for the *cox1* coding gene^[^^22^^]^. The total volume of 25 µl was selected for PCR reaction (one unit of Taq DNA polymerase, 2 µl of MgCl_2_, 0.5 µl for each forward and reverse primers, 1 µl of DNA, 2.5 µl of buffer 10×, 2 µl of dNTP, and 16.5 µl of distilled water (Table 1). After preparing the PCR mixture, the thermocycler program was set to 35 cycles. The amplified fragments were electrophoresed on 1.5% agarose gel and then visualized by DNA-safe stain (Cinaclone, Iran).

**DNA sequencing method**

 All sequences were compared pairwise with each other, and the sequences were deposited in the GenBank using the program nucleotide BLAST of the NCBI (http://blast.ncbi.nlm.nih.gov/Blast.cgi). Equal length for each sequence was trimmed with Bioedit software v.7.2 (https://bioedit.software.informer.com/ 7.2/), and global sequence alignments were performed using the ClustalW algorithm. A phylogenetic analysis was carried out on the sequence data obtained in the 

**Table 1:** PCR conditions used in the present study

**Test stage**	**Temperature (** **°** **C)**	**No. of ** **cycles**	**Time**
Primary denaturation	95	1	5 min
			
Denaturation	94	35	45 s
Annealing	51		35 s
Extension	72		45s
			
Final extension	72	1	10 min

**Table 2:** Cocktail staining for visualizing each enzyme in polyacrylamide gel

**Enzyme**	**Buffer pH**	**Substrate**	**Coenzyme**
MDH	8.0	Malic acid	NAD
G6PD	7.4	G6P	NADP
ICD	7.0	Isocitrate	NADP
NH1	7.4	Inosine	Not needed
ME	7.4	Malic acid	NADP

Tris-HCl (0.3 mol) was the buffer used in this study.

present study, and the data were compared with other species/genotypes of *Echinococcus*. The best-fit nucleotide substitution model and phylogenetic tree were generated using Mega 6 software (https://www. megasoftware.net).

**Enzyme extraction**

 The method of comparing isozymes was performed based on approaches used previously in 2020^[^^23^^,^^24^^]^. The sediments of samples were thoroughly mixed with equal volumes of enzyme stabilizers (2 mM of dithiothreitol, aminocaproic acid, and EDTA solution). The samples were freezed and thawed at -196 °C and 25 °C, respectively for 10 times and then transferred to 0.5-ml tubes. The samples were then centrifuged at 15,000 ×g at 4 °C for 80 min, and the supernatant solution containing water-soluble proteins was kept at -70 °C until use.

**Isoenzyme electrophoresis**

 The discontinuous polyacrylamide gel electro-phoresis method was applied to perform the isoenzyme electrophoresis by vertical electrophoresis^[^^25^^]^. Electrophoresis was performed using the stacking gel of 4% and separating gel of 8%. In the present study, we assessed the activities of five enzymatic systems, including G6PD, ME, ICD, MDH, and NH1. The RF or relative migration distance, the number, and frequency of isoenzyme banding patterns or zymodeme of each genotype were determined and compared with each other. To determine the RF value of each band, we divided the distance traveled by the isoenzyme band from the origin by the length of the gel. Cocktail staining for visualizing each enzyme in polyacrylamide gel is shown in Table 2. 

## RESULTS

 A total of 32 CE cyst isolates were isolated from 8 humans and 24 animals (sheep, cattle, and camels). The collected samples were characterized by PCR and then sequencing as *E. granulosus s.s.* (24 isolates) and *E. canadensis* (8 isolates) genotypes (Table 3).

**Results of PCR for** ***cox1*** **primers**

**Fig. 1:**
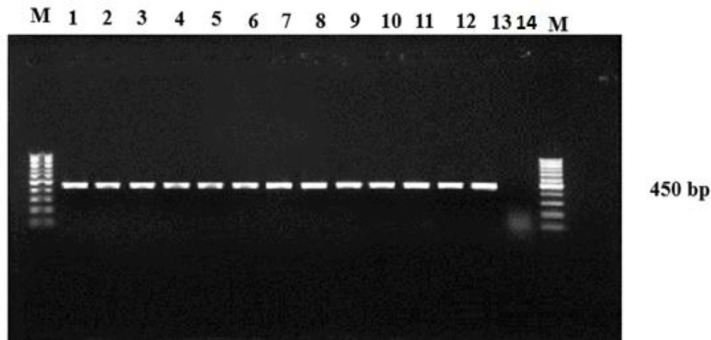
Agarose gel electrophoresis showing the PCR product analysis of *cox1* gene in *E. granulosus s.s. *M, marker (100 bp); lanes1-13, positive *cox1* gene at the 450-bp PCR product; lane 14, negative control

Agarose gel electrophoresis for *cox1* primers is depicted in Figure 1.

**Results of DNA sequencer technique and phylogenetic tree analysis**

**Fig 2:**
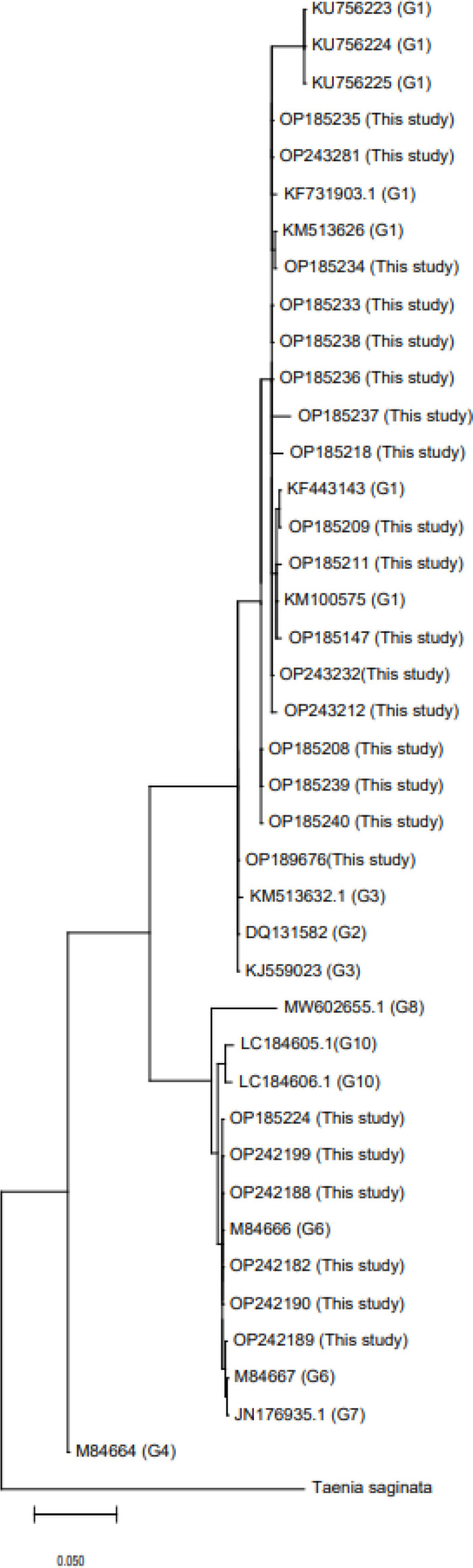
Molecular phylogenetic tree of mitochondrial *cox1* region of *E. granulosus* *s.s. *and *E. canadensis* (G6/G7) genotypes. 1000 bootstrap replicates

Samples were sent to Takapouzist Company for identifying the DNA sequence. The results showed that *E. granulosus s.s.* (G1) was present in 75% of the samples, and 25% were *E. canadensis* (G6). The phylogenetic tree was constructed using the evolutionary distances computed using the Maximum Composite Likelihood method (UPGMA tree) in the MEGA 6.0 version (Fig. 2 and Table 4).

**Table 3:** Different *E. granulosus* genotypes based on location, tissue, and type of host

**Variable**	**Frequency**	***E. granulosus*** ***s.s.*** **(G1-G3) genotype**	***E. canadensis*** **(G6/G7) genotype**
Location of host			
Shiraz	16	16	-
Tehran	4	4	-
Ilam	4	4	-
Birjand	8	-	8
			
Tissue of host			
Liver	22	16	6
Lung	10	8	2
			
Type of host			
Human	8	8	-
Sheep	8	8	-
Cattle	8	8	-
Camel	8	-	8`
			
Total	32	24	8

**Isoenzyme patterns** **of G6PD, ME, MDH, NH1, and ICD**

**Fig. 3:**
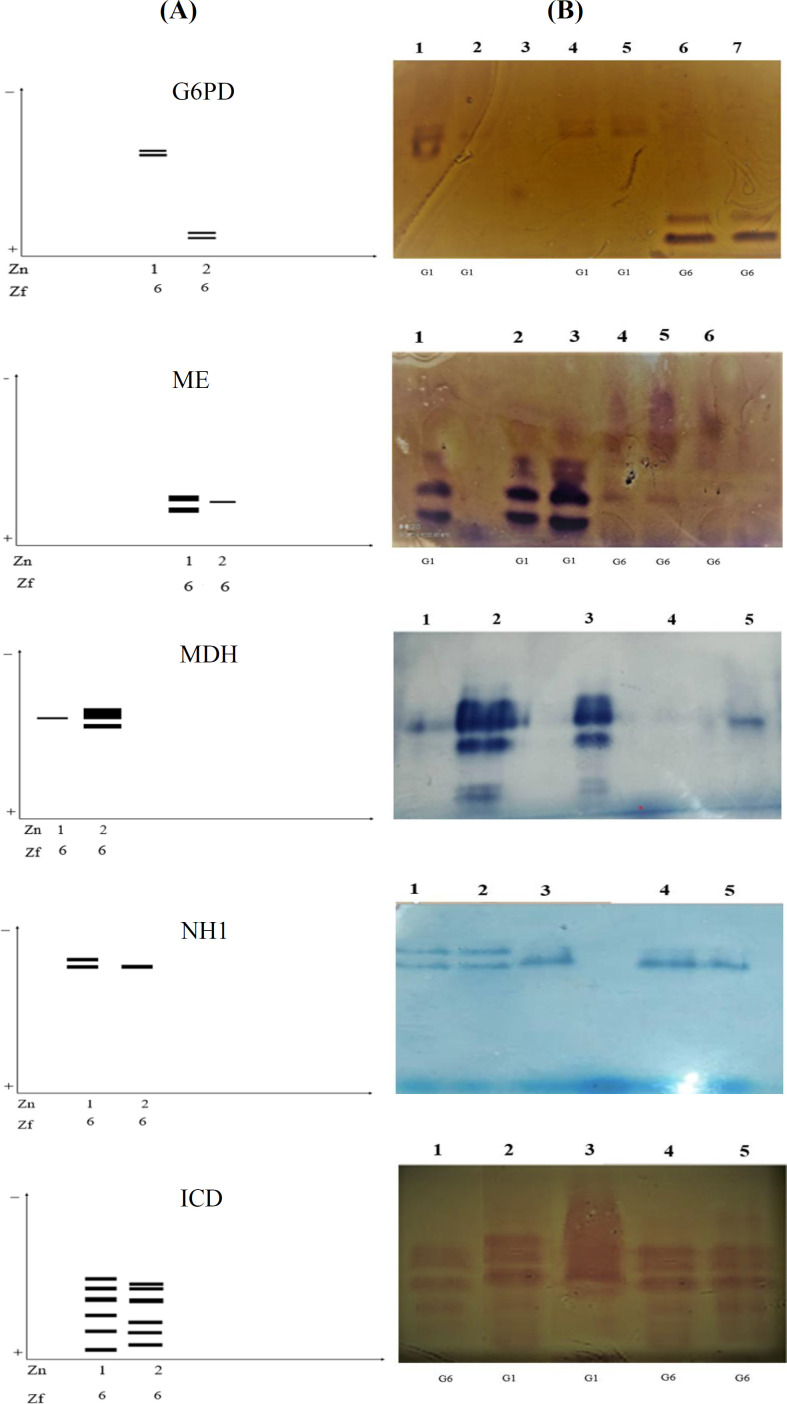
(A) Zymodeme of G6PD, ME, MDH, NH1, and ICD obtained from *E. granulosus s.s.* and *E. canadensis* genotypes; (B) the isoenzyme pattern illustration of G6PD, ME, MDH, NH1, and ICD in the present study. Dark bands shown in the first Figure (G6PD) are our zymodeme samples. Zn, zymodeme number; Zf, zymodeme frequency

 The isoenzyme patterns of G6PD, ME, MDH, and NH1 revealed two zymodemes. Zymodeme 1 was observed in 24 isolates, all of which were *E. granulosus s.s.* genotype, and zymodeme 2 was detected in 8 isolates that were* E. canadensis* genotype (Fig. 3). The ICD isoenzyme pattern also demonstrated two zymodemes, zymodeme 1 in 26 isolates (81.25%) and zymodeme 2 in 6 (18.75%) isolates that were *E. granulosus* *s.s*. and *E. canadensis* genotypes respectively (Fig. 3E). The number of bands and RF in each enzymatic system is shown in Table 5.

## DISCUSSION

The present study investigated the isoenzyme pattern and molecular characterization of the *E. granulosus s.l. *larval stage in different parts of Iran. 

The result showed that *E. granulosus* *s.s. *and *E. canadensis* genotypes had entirely different isoenzyme patterns in four enzyme systems (NH1, G6PD, MDH, and ME) used in this study. However, in the ICD enzyme system, it was impossible to differentiate between *E. granulosus* *s.s.* from *E. canadensis* strains. In the *E. granulosus* *s.s.* strain, MDH and NH1 revealed a two-band pattern, whereas the ME enzyme had a single main band. This trend for *E. canadensis* strain was reverse. Both genotypes studied in ICD and G6PD enzyme systems had the same number of bands with different RF values. The provided isoenzyme data represent a high degree of homogeneity within the studied strains (*E. granulosus s.s. *and *E. canadensis* strains). In this research, the isolates of each species showed similar iso-enzymatic patterns, and the studied enzymatic systems clearly showed the difference between the two species. Our results highlight the importance of using several different criteria when attempting to fully characterize an intra-strain differentiation of *E. granulosus* in any geographical locality and from a particular intermediate (or definitive) host. These criteria include ecological, immunological, morphological, biochemical, isoenzymatic, and in vitro developmental studies^[^^26^^]^. The study performed by Haag et al.^[27]^, revealing *E. granulosus* intra-strain polymorphisms, reported limited or no variation within the four studied isolates; this finding is consistent with the isoenzyme pattern reported by other researchers^[^^25^^,^^27^^]^. 

The results obtained in the current study regarding the ability of the zymodeme technique to differentiate between *E. granulosus* genotypes are in linem with those reported by other studies in different geographical areas^[^^26^^,^^28^^,^^29^^]^. Turčeková et al.^[^^30^^]^ proved the suitability of GPI and MDH enzymes for discriminating G7 and G1 of *E. granulosus* strains. Hosseini et al.^[^^31^^]^ investigated the isozyme pattern of G6PD and ICD in *E. granulosus* *s.s.* native to Iran and detected two different strains, sheep-dog (genotype G1) and camel-dog (genotype G6) of this parasite^[31]^. Siles-Lucas and Cuesta-Bandera^[^^32^^]^ showed that the SDS-PAGE technique for differentiating between the various *E. granulosus* strains was inappropriate. However, we found out that zymogram analysis of *E. granulosus* extracts using discontinuous SDS method was suitable to identify different *E. granulosus* strains.

We are actually examining a parasite's phenotypic characteristics when we compare the isoenzymes of various parasite genotypes, and because these phenotypic characteristics accurately depict the genotype, the results can be taken to be in agreement with one another. In a previous electrophoretic study, Le Riche and Sewell^[^^33^^]^ suggested that GPI isoenzyme profiles of *E. granulosus* belonging to UK sheep and cattle are very similar. However, these patterns are different from those produced by hydatid material obtained from two infected Nigerian camels. Isoenzyme technique as a relatively inexpensive method is still a marker with perfectly-known Mendelian inheritance, which permits multilocus analysis and is applied for most living organisms. Population genetics and phylogenetic research benefit from the use of two different types of genetic markers since they can examine various regions of the genome and have different evolutionary patterns^[^^34^^]^. In addition to molecular and pharmacological approaches, proteomic approaches play a significant role in identifying several excretory-secretory proteins. The ability to fully comprehend the connection between Echinococcus and its host and the mechanisms mediated by them is made possible by the identification of the proteins present in hydatid fluid. Many of these proteins may only or mostly be expressed by specific isolates and species; therefore, proteomic information is also very useful in interpreting the results of molecular and biochemical investigations^[^^35^^]^. 

The method of determining isoenzyme characteristics in many microorganisms, such as Leishmania parasite species, has been proposed as a gold standard and as a repeatable and very accurate phenotypic method for identifying other infectious disease agents, which is of interest to researchers and scientists. Despite the emergence of new techniques, such as proteomics and genome-based methods, the mentioned phenotypic method still maintains its value. However, our study has some limitations, including the small number of enzyme systems examined and the lack of *E. granulosus* larval stage isolates from other intermediate hosts, such as goat, in Iran.

The findings of the present study show that the* E. granulosus s.s.* and the* E. canadensis* genotypes have entirely different isoenzyme patterns in the enzymes of NH1, G6PD, MDH, and ME. The importance of isoenzyme method increases when the difference between two genotypes is clearly shown by using a limited number of enzyme systems. Such precision in distinguishing two different organisms that are macroscopically similar has a great advantage in biology.

**Table 4:** Information about sequences used for phylogenetic analysis

**Accession number**	***E. granulosus *** **genotype**	**Reference**	**Country**	**Accession number**	***E. granulosus *** **genotype**	**Reference**	**Country**
OP185147	G1	This study	Iran	KM513632	G3	^[^^36^^]^	Iran
OP185208	G1	This study	Iran	DQ131582	G2	^[^^37^^]^	Portugal
OP185209	G1	This study	Iran					
OP189676	G1	This study	Iran	KT988115	G5	^[^^38^^]^	Iran
OP185211	G1	This study	Iran	KJ559023	G3	^[^^39^^]^	China
OP243212	G1	This study	Iran	OP185233	G6	This study	Iran
OP185218	G1	This study	Iran	OP242182	G6	This study	Iran
OP185224	G1	This study	Iran	KT988117	G6	^[^^38^^]^	Iran
OP243232	G1	This study	Iran	M84666	G6	^[^^42^^]^	Australia
OP185234	G1	This study	Iran	KT988116	G6	^[^^38^^]^	Iran
OP185235	G1	This study	Iran	M84667	G6	^[^^40^^]^	Australia
OP185236	G1	This study	Iran	OP242188	G6	This study	Iran
OP185237	G1	This study	Iran	OP242189	G6	This study	Iran
OP185238	G1	This study	Iran	OP242190	G6	This study	Iran
OP185239	G1	This study	Iran	OP242199	G6	This study	Iran
OP185240	G1	This study	Iran	OP185224	G6	This study	Iran
OP243281	G1	This study	Iran	OP185233	G6	This study	Iran
KU756223	G1	^[38]^	Iran	M84664	G4	^ [^^40^^]^	Australia
KU756225	G1	^[38]^	Iran	KF731903	G1	^[^^41^^]^	Iran
KU756224	G1	^[38]^	Iran	KF443143	G1	^[^^42^^]^	Iran
KM513626	G1	^[36]^	Iran	KT988113	G1	^[^^38^^]^	Iran
KM100575	G1	^[43]^	Turkey	M84668	*E. multilocularis*	^[^^40^^]^	Australia
Ab107243	Outgroup	^[45]^	Nepal	MW736596.1	G7	^[^^44^^]^	Peru

**Table 5:** General information of two different zymodemes in the present study

**Enzyme system**		**Zymodem 1** **(** ***E. granulosus s.s. *** **genotype)**					**Zymodem 2** **(** ***E. canadensis*** ** genotype)**			
		**No. of isolates**	**Accession** **no.**	**No. of bands**	**RF**		**No. of isolates**	**Accession** **No.**	**No. of bands**	**RF**
MDH		6	OP185238 OP185239 OP185240 OP243281 OP185235 OP185236	1	0.60		6	OP185233 OP242188 OP242189 OP242190 OP242199 OP185224	2	0.51, 0.68
										
G6PD		6	OP185147 OP185208 OP185209 OP189676 OP185211 OP243212	2	0.6, 0.62		6	OP185233 OP242188 OP242189 OP242190 OP242199 OP185224	2	0.15, 0.2
										
ICD		6	OP243212 OP185218 OP185224 OP243232 OP185234 OP185237	6	0.58, 0.54, 0.43, 0.34, 0.22, 0.17		6	OP185233 OP242188 OP242189 OP242190 OP242199 OP185224	6	0.65, 0.56, 0.52, 0.35, 0.31, 0.26
										
NH1		6	OP185237 OP185238 OP185239 OP185209 OP189676 OP185211	2	0.77, 0.69		6	OP185233 OP242188 OP242189 OP242190 OP242199 OP185224	1	0.73
										
ME		6	OP185239 OP185211 OP243281 OP185218 OP243232 OP185235	2	0.17, 0.31		6	OP185233 OP242188 OP242189 OP242190 OP242199 OP185224	1	0.26

## DECLARATIONS

### Acknowledgments

This article has been derived from a Ph.D. thesis (number: 20037) at Shiraz University of Medical Sciences, Shiraz, Iran. We would like to thank the cooperation of all the research centers and units.

### Ethical statement

The protocol of this study was approved by the Ethics Committee of the Shiraz University of Medical Sciences, Shiraz, Iran (ethical code: IR.SUMS.REC. 1399.378). 

### Data availability

The datasets used and analyzed during the current study are available from the corresponding author on reasonable request.

### Author contributions

MD: formal analysis and investigation, methodology, and writing original draft preparation; SMS: conceptualization and Writing-review and editing; RS: formal analysis and investigation; AV: methodology; AR: methodology; GRH: conceptualization, writing-review and editing, funding acquisition, resources, and supervision.

### Conflict of interest

None declared.

### Funding/support

No fund received.
